# Molecular Characterization of Adult-Type Lower-Grade Glioma (WHO Grade 1–3) with Targeted Next-Generation Sequencing: A Retrospective, Single-Institution Experience

**DOI:** 10.3390/jcm15010053

**Published:** 2025-12-21

**Authors:** Maurizio Pinamonti, Maurizio Polano, Giacomo Cester, Federico Saturno Spurio, Erik Roman-Pognuz, Maja Ukmar, Michele Dal Bo, Fabrizio Zanconati, Leonello Tacconi, Antonio Meola

**Affiliations:** 1 Pathology Unit, Department of Medicine, Surgery and Health Sciences, University Hospital and Health Services of Trieste—ASUGI, University of Trieste, Strada di Fiume 447, 34149 Trieste, Italy; fabrizio.zanconati@asugi.sanita.fvg.it; 2 Experimental and Clinical Pharmacology Unit, Centro di Riferimento Oncologico di Aviano, Istituto di Ricovero e Cura a Carattere Scientifico, 33081 Aviano, Italy; mpolano@cro.it (M.P.); mdalbo@cro.it (M.D.B.); 3 Radiology Unit, Department of Medicine, Surgery and Health Sciences, University Hospital and Health Services of Trieste—ASUGI, University of Trieste, Strada di Fiume 447, 34149 Trieste, Italy; giacomo.cester@asugi.sanita.fvg.it (G.C.); maja.ukmar@asugi.sanita.fvg.it (M.U.); 4 Neurosurgery Unit, Department of Medicine, Surgery and Health Sciences, University Hospital and Health Services of Trieste—ASUGI, University of Trieste, Strada di Fiume 447, 34149 Trieste, Italy; federico.saturnospurio@asugi.sanita.fvg.it (F.S.S.); leonello.tacconi@asugi.sanita.fvg.it (L.T.); 5 Intensive Care Unit, Department of Medicine, Surgery and Health Sciences, University Hospital and Health Services of Trieste—ASUGI, University of Trieste, Strada di Fiume 447, 34149 Trieste, Italy; erik.romanpognuz@asugi.sanita.fvg.it

**Keywords:** lower-grade glioma, next-generation sequencing, IDH1 gene, brain tumor

## Abstract

**Background/Objectives**: The 2021 WHO Classification of Central Nervous System (CNS) tumors emphasizes the integration of molecular data with histopathological features. Lower-grade gliomas (LGGs) represent a heterogeneous group of neoplasms with variable clinical behavior. This study aimed to explore the molecular landscape of a single-institution series of LGGs using targeted next-generation sequencing (NGS). **Methods**: Eleven adult patients diagnosed with LGG between 2015 and 2024 at Cattinara University Hospital (Trieste, Italy) were retrospectively analyzed. DNA and RNA were extracted from formalin-fixed, paraffin-embedded (FFPE) tissue and analyzed using the TruSight Oncology 500 panel (Illumina). Mutational, amplification, and transcriptomic profiles were evaluated. **Results**: IDH1 mutations were the most frequent alteration (75%), commonly co-occurring with TP53 and ATRX mutations, consistent with the canonical IDH-mutant astrocytoma profile. CDK4 amplification was found in four cases, while MYCN amplification and MET amplification were each identified in isolated cases. Two diffuse IDH-wild-type gliomas displayed aggressive clinical courses and shorter survival, and one was reclassified as glioblastoma (grade 4) based on EGFR amplification. The transcriptome analysis revealed heterogeneous expression signatures and distinct clustering of IDH1/ATRX-mutant tumors. **Conclusions**: Targeted NGS confirmed the key molecular features of diffuse gliomas and enabled precise WHO 2021 classification even in archival FFPE samples. Despite the exploratory nature of the analysis on a small population, the study underscores the biological and transcriptional heterogeneity of LGGs and highlights the limitations of tumor-only sequencing approaches. Broader genomic profiling and matched normal controls are warranted to refine the interpretation of rare or non-canonical variants.

## 1. Introduction

Glioblastoma is the most common glial neoplasm in adults, and—after meningiomas—it is also the most common intracranial tumor [1]. Lower-grade gliomas (LGGs) are generally less common and represent a heterogeneous group of brain tumors with variable prognoses and biological behavior. In fact, prognosis depends upon many factors, including their location within the central nervous system (CNS), the histological grade, the tendency toward local infiltration, and certain genetic abnormalities. The grading system for CNS tumors is traditionally mostly based on morphological criteria, including, but not limited to, mitotic activity, microvascular proliferation, and necrosis [2]. More recently, several molecular analyses have become instrumental for a complete and accurate diagnosis. Thus, the term “integrated” diagnosis refers to the combination of morphological information along with genetic–molecular criteria [3].

The advent of modern next-generation sequencing (NGS) techniques allowed for the identification of molecular biomarkers with diagnostic and prognostic relevance, leading to a revision of the WHO classification criteria of CNS tumors [4,5]. Among the most well-known gene alterations of brain tumors, there are mutations of isocitrate dehydrogenase (IDH) 1 and 2 gene, whole arm deletion of chromosome arms 1p and 19q, mutations of the Telomerase Reverse Transcriptase (TERT) promoter, amplifications and mutations of Human Epithelial Growth Factor Receptor (EGFR), and mutations of B-Raf proto-oncogene, serine/threonine kinase (BRAF) [6]. Despite these advances, the molecular landscape of LGGs remains incompletely characterized, especially regarding less prevalent gene variants (e.g., RET, FGFR4). Targeted next-generation sequencing (NGS) approaches applied to FFPE material have emerged as a valuable, cost-effective tool for multiplex genomic profiling in routine diagnostic workflows [7]. Combined DNA/RNA sequencing is particularly attractive, as it expands detection to gene fusions and transcripts relevant for emerging targeted therapies.

The aim of this exploratory, single-institution study is therefore to assess the feasibility, molecular spectrum, and potential clinical relevance of targeted NGS (DNA+RNA) in a series of adult-type lower-grade gliomas, contributing further real-world evidence to their integrated characterization.

## 2. Materials and Methods

### 2.1. Study Population

A cohort of patients affected with LGGs WHO grade 1–3 and operated at the “Cattinara” University Hospital of Trieste (ASUGI) from January 2015 to January 2024 was extracted from the pathology management software APSYS (version 10.00.06, Insiel corp., Trieste, Italy).

**Figure 1:**
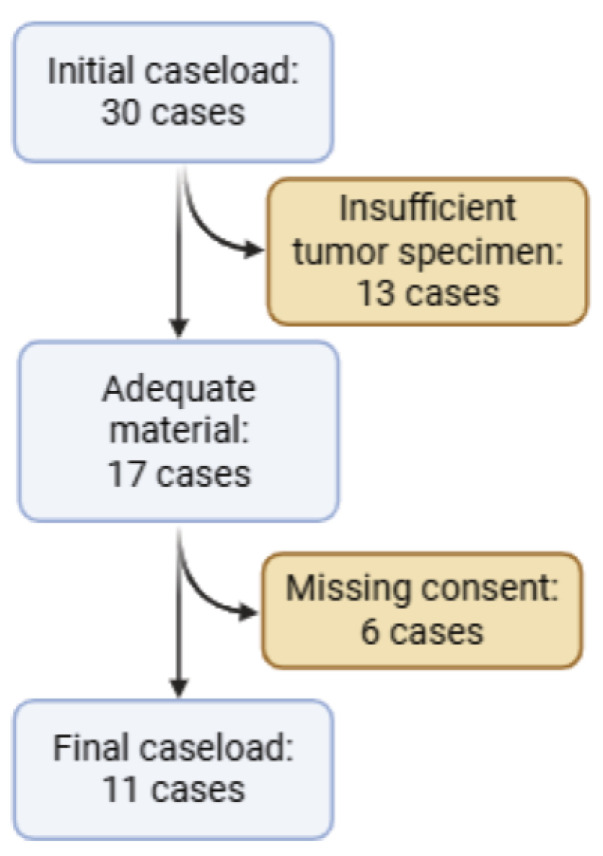
Case flow diagram documenting initial and final case load and reasons for exclusion.

The study cohort included adult patients (aged 18 years and above) who underwent either surgical resection or biopsy followed by any other adjuvant treatments for LGG at our institution. Patients were excluded if informed consent for research and publication could not be obtained or if the pathology specimen for histological and molecular analysis was deemed degraded or insufficient (Figure 1).

Patients‘ data were retrospectively collected. Demographic data included gender, age at diagnosis, use of steroids, presence of symptoms and seizures at the time of diagnosis, progression-free survival (PFS), and overall survival (OS). PFS was defined as the time from surgery to first progression. OS was defined as the time from diagnosis to death or to the last follow-up.

Histological and molecular diagnosis was conducted according to the 2021 WHO classification of the Tumors of the CNS [5]. The histological analysis of patients operated on before 2021 was reviewed by two experienced pathologists (MP, FZ). Collected histological data included histological type, histological grade, IDH mutational status, and MGMT methylation.

### 2.2. Next-Generation Sequencing (NGS)

Genomic DNA and total RNA were extracted from formalin-fixed, paraffin-embedded (FFPE) (5-uM) tumor tissue using the AllPrep DNA/RNA FFPE Kit (Qiagen, Hilden, Germany). Tumor areas were macro-dissected when needed to ensure ≥20% neoplastic cellularity. DNA and RNA input quantities followed Illumina’s recommended specifications for the TruSight Oncology 500 (TSO500) assay and the TruSeq Total Stranded RNA protocol, thereby ensuring adherence to validated input requirements and optimal performance of both DNA- and RNA-based analyses from FFPE material. The TSO500 assay is a clinically validated hybrid-capture platform, with extensive single- and multi-center validation documenting high analytical performance for SNVs/indels, CNVs, and fusions [8,9].

DNA libraries were prepared with the TruSight Oncology 500 (TSO500) DNA Kit (Illumina, San Diego, CA, USA) following the manufacturer’s instructions, as previously described [10]. DNA data were processed locally using the Local Run Manager (LRM) TruSight Oncology 500 Analysis Module v2.2, executed within the official Illumina Docker environment. This pipeline constitutes the validated analytical backbone of the TSO500 assay and includes UMI-aware preprocessing, read stitching, and indel realignment performed by the Gemini modules, followed by alignment to the hg19 reference genome using BWA-MEM. Somatic SNVs and indels were called using the Pisces variant caller, with post-processing and quality refinement applied through the Pepe module. Variants were retained according to Illumina’s validated thresholds for FFPE tumor-only workflows, which require a minimum depth of 100×, a variant allele frequency of at least 5%, sufficient mutant read support—including at least one stitched mutant read for indels—and the absence of strand- or read-type biases. Additional filtering excluded positions with excessive polymorphic burden, such as high-frequency COSMIC loci, and removed variants located in assay blacklist or low-mappability regions while applying FFPE artifact-suppression rules. Copy number alterations were inferred using the CRAFT algorithm, which normalizes depth against the internal panel of normals and applies gene-specific thresholds for gains, amplifications, and deletions. Microsatellite instability was assessed using the entropy-based MSI model integrated in TSO500 v2.2, which requires at least 40 evaluable loci for classification. Variant annotation was performed via the Illumina Nirvana engine, incorporating gnomAD v2.1, COSMIC v84, ClinVar (4 February 2019), dbSNP v151, and Ref-Seq/Ensembl transcript models. This workflow, which mirrors the structure and analytical logic described by Dal Bo et al., ensured a standardized and internally consistent approach to DNA variant detection and interpretation across all cases.

RNA-seq libraries were then prepared following the TruSeq Total Stranded RNA protocol (Illumina), a validated method for generating high-fidelity, strand-specific libraries that preserve transcript orientation and improve quantification accuracy. Only RNA samples with DV200 ≥ 30%, in accordance with Illumina’s recommendations for FFPE-derived RNA, were considered suitable for library preparation. The libraries were sequenced on the Illumina NextSeq 550 platform (Illumina, San Diego, CA, USA), achieving a depth of 50 million paired-end reads (2 × 100 bp) per sample, ensuring sufficient coverage for comprehensive transcriptome analysis.

Reads were aligned and quantified against the GENCODE v19 reference transcriptome using STAR (v2.7.10a), and transcript abundance was subsequently quantified with RSEM (v1.3.3), as implemented in the nf-core/rnaseq pipeline (v3.17.0). This workflow ensures accurate and reproducible estimation of gene and transcript expression levels. Sequencing-quality metrics were assessed with FastQC (v0.11.x), including evaluations of per-base sequence quality, GC content, duplication rates, and potential adapter contamination, thereby confirming the suitability of the datasets generated for downstream analyses.

Gene fusions were identified using GeneFuse (v0.8.0), which reconstructs candidate fusion junctions by integrating split-read alignments, spanning-read evidence, breakpoint orientation, and local sequence complexity. GeneFuse’s targeted filtering strategy, designed to limit false-positive calls in FFPE-derived material, allows for high-confidence characterization of rearrangements detectable within the sequencing depth and library preparation constraints applied in this study [11].

### 2.3. Treatment Strategy

All patients underwent craniotomy for either tumor resection or neuronavigation-guided brain biopsy. Whenever feasible, gross-total resection with neuronavigation and neurophysiological monitoring was the treatment of choice. When tumor resection was not feasible or declined by the patient, a brain biopsy was offered.

After surgical treatment and histological diagnosis, patients were evaluated by an experienced team of oncologists and radiation oncologists. Adjuvant treatment was recommended on an individual basis, including concomitant chemoradiation according to the Stupp protocol, Procarbazine, and Lomustine or fractionated brain radiotherapy alone [12,13].

### 2.4. Radiological Follow-Up Strategy

All patients underwent brain magnetic resonance imaging (MRI) with gadolinium contrast before surgery and then within 3 months after surgery to estimate the residual tumor volume, if any. After the first post-operative MRI, the schedule of subsequent scans was individualized depending upon the estimated risk of tumor recurrence.

The volumetric analysis was performed by two experienced academic neuroradiologists (GC and MU), along with a neurosurgeon (AM). The quantitative assessment of initial tumor volume and post-operative residual was conducted on the preoperative and postoperative fluid-attenuated inversion recovery (FLAIR) sequences, by using the Element segmentation software (BrainLab™, Munich, Germany). Based on tumor residual, resection was classified as gross-total resection (GTR) if above 98% of the initial tumor volume, sub-total resection (STR) if between 90% and 98%, and partial resection (PR) if below 90%. The timing of further MRI follow-up was individualized depending on the adjuvant treatment, recurrence, and patients’ status. Tumor progression was diagnosed according to RANO Guidelines [14].

Given the size of the small size of the cohort, formal survival analysis was not performed.

## 3. Results

The initial caseload included 30 cases. However, several cases were excluded because of two main reasons: first, insufficient or inadequate tumor specimen for molecular analysis (13 cases), and second, for missing consent for research and publication (6 patients) (Figure 1). The final cohort included 11 patients. The characteristics of the case series are shown in Table 1. Detailed clinical, histopathological, molecular, and follow-up information for each case is provided in Supplementary Table S1.

The original pathology diagnoses of lesions were the following: five astrocytoma IDH-mutant, two oligodendroglioma IDH-mutant and 1p/19q co-deleted, one pilocytic astrocytoma, one ganglioglioma, and two diffuse gliomas with morphological features consistent with astrocytoma grade 2, with IDH wild type. One of these (case 10) had a diagnosis formulated prior to the fifth edition of the WHO classification and, therefore, had been diagnosed as fibrillary astrocytoma, grade 2, regardless of IDH status, without any further molecular investigations being performed. The second one (case 5) was a case subject to external review and molecular investigations without meeting the criteria currently required for the diagnosis of glioblastoma [5,15] and was therefore concluded as “IDH-wild type diffuse astrocytic neoplasm with low-grade morphology, grade 2”. The median age at diagnosis of patients was 40, ranging from a minimum of 19 to a maximum of 75. Most lesions (8; 72.73%) were located in the frontal lobe, while the others were mostly in the parietal lobe (2; 18.18%) and the cerebellum (1; 9.09%). Eight patients presented with symptoms, while in three, the finding was incidental. Seizures occurred in seven patients (63.64%).

All patients underwent open craniotomy for tumor resection, except one patient who underwent a brain biopsy. GTR was achieved in six cases (54.55%), while STR was achieved in four cases (36.36%). Five patients (45.45%) did not receive any adjuvant treatment. Stupp regimen was adopted in three cases (27.27%), PCV protocol in one case (9.09%), and radiotherapy alone in two cases (18.18%).

Six patients had disease progression, with a median PFS of 17.5 months (range: 1.8–50.3). Two patients died, with a median OS of 11.38 months. Both deceased patients had a diagnosis of morphologically low-grade, IDH-wild-type, diffuse glioma.

Survival outcomes were descriptive only, as the cohort was not powered for clinical association analyses. Due to the exploratory nature of the study and the high exclusion rate, the final analytic cohort may not be fully representative of the broader LGG population.

Molecular analyses were successfully performed on all cases. Table 2 lists cases carrying mutations interpreted as pathogenic, likely pathogenic, drug-putative, risk factor, or of uncertain significance/with conflicting interpretations. Table 3 lists the genetic amplifications found in the case series.

In our cohort, the most recurrent alteration was represented by IDH1 mutations, detected in approximately 75% of cases. Concomitant mutations in ATRX and TP53 were frequently observed, consistent with the canonical molecular profile of IDH-mutant astrocytomas. A limited subset of cases harbored alterations in the TERT promoter, which have been associated with an unfavorable prognosis in diffuse gliomas. Additional variants were identified in genes such as CHEK2, FGFR3, TET2, ASXL1, and ZFHX3, each occurring in single cases. Variants in RET and FGFR4 (e.g., RET p.G691S, FGFR4 p.G388R) were excluded from the somatic landscape, as they represent common germline polymorphisms with high-population allele frequencies.

**Figure 2:**
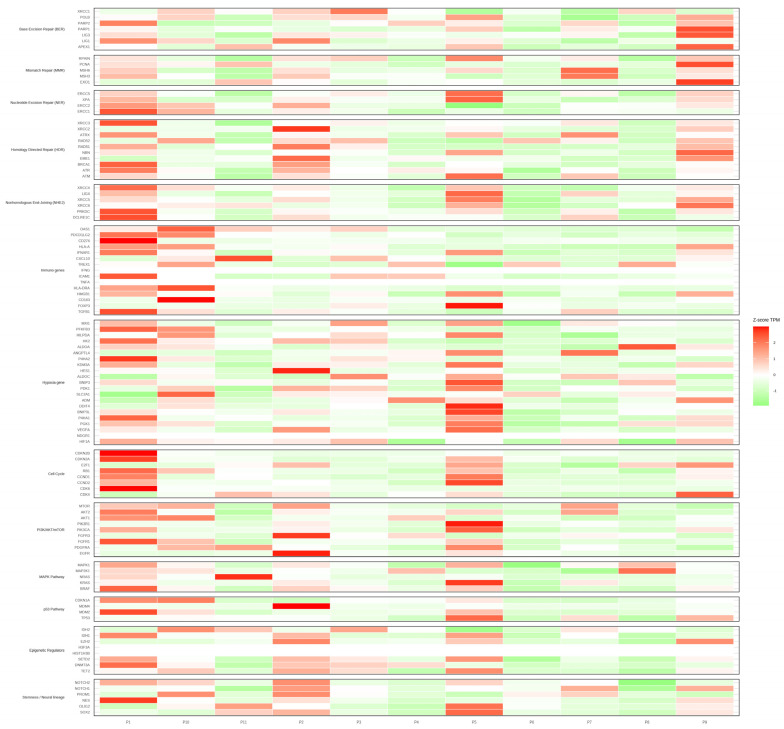
Gene expression heatmap of the study cohort illustrating the transcriptional profiles across all tumor samples. Rows represent genes and columns represent individual cases. Distinct clustering patterns are evident, with IDH1/ATRX-mutant tumors showing a more homogeneous expression signature, while other cases exhibit greater heterogeneity. Color intensity reflects relative expression levels after normalization, highlighting pathway-specific transcriptional differences.

**Figure 3:**
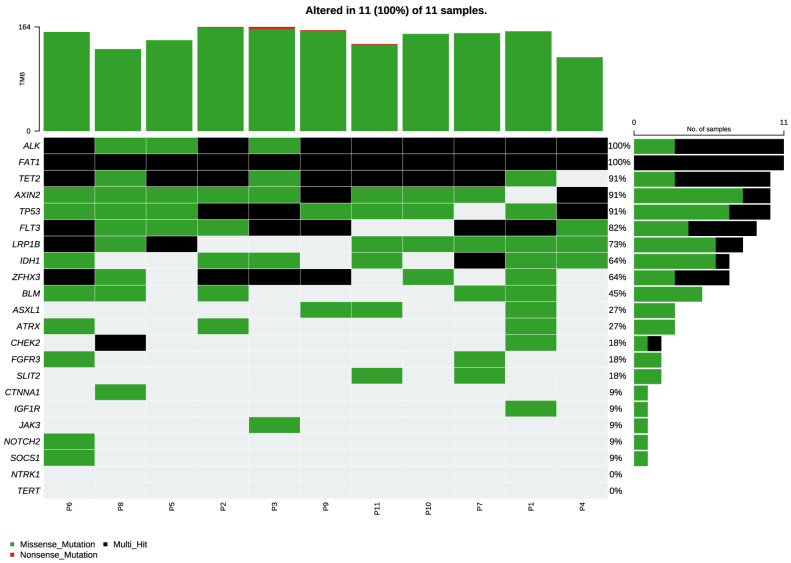
Somatic mutation landscape of the targeted gene panel across 11 tumor samples. The visualization includes every variant called by the bioinformatic pipeline, such as missense mutations, multi-hit events, and alterations that may represent germline polymorphisms, artifacts, or non-driver (passenger) events. For analytical rigor, the manuscript focuses exclusively on high-confidence somatic variants and bona fide oncogenic drivers, while common polymorphisms and low-confidence calls are not considered in the biological interpretation. Genome-wide coverage metrics for all high-confidence somatic variants are reported in Supplementary Table S5.

Gene expression profiling across the cohort revealed a heterogeneous transcriptional landscape, as illustrated in the heatmap (Figure 2). Distinct clusters of samples were observed, reflecting differential expression patterns in key oncogenic and tumor suppressor pathways. Notably, cases harboring canonical alterations such as IDH1 and ATRX tended to segregate together, suggesting a transcriptional signature associated with the IDH-mutant astrocytoma subgroup. Additional heterogeneity was evident among samples lacking these alterations, where expression variability involved genes implicated in cell cycle regulation, DNA repair, and receptor tyrosine kinase signaling (Figure 3). RNA-seq quality control metrics, including raw and post-trimming read counts, GC content, and trimming rates for all samples, are reported in Supplementary Table S2. RNA-sequencing data analyzed with GeneFuse (v0.8.0) did not reveal recurrent fusions across the cohort. In a single case, a putative ALK–ROS1 rearrangement was detected and nominally supported by six split reads. However, inspection of the underlying alignments showed features incompatible with a bona fide fusion event, including inconsistent breakpoint orientation, absence of spanning-read support, and low-complexity sequence at the junction—patterns characteristic of FFPE-related chimeric artifacts and below the confidence thresholds typically expected for GeneFuse high-confidence calls. Moreover, ALK and ROS1 rearrangements had already been assessed during the initial diagnostic evaluation using an original diagnostic work-up using a clinically validated fluorescence in situ hybridization (FISH) assay performed at an external accredited pathology laboratory, which demonstrated no evidence of ALK gene rearrangement. Taken together, the limited and discordant read evidence, the known susceptibility of FFPE RNA to generate artefactual chimeras, and the negative orthogonal clinical testing supported classification of this event as a false positive. Accordingly, it was excluded from the final set of somatic alterations. Full fusion-detection outputs and read-level inspection of the ALK–ROS1 artifact are provided in Supplementary Tables S3 and S4.

**Figure 4:**
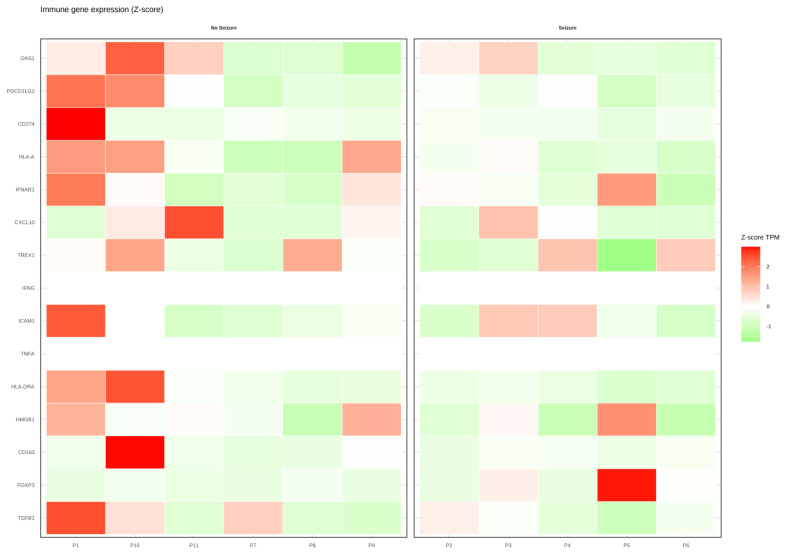
Heatmap showing the expression levels of immune-related genes (Z-score TPM) in patients without seizures (“No Seizure”) and with seizures (“Seizure”). Each row represents an immune gene involved in innate or adaptive responses, and each column corresponds to an individual patient. Gene expression values are scaled using Z-scores to highlight relative differences across samples. Red indicates higher expression, while green denotes lower expression. The heatmap reveals distinct immune-expression patterns between patients with and without seizures.

Analysis of immune-related gene expression (Figure 4) revealed heterogeneous transcriptional profiles across the cohort. In group 0, several immune checkpoint and pro-inflammatory genes, including PDCD1LG2 (PD-L2), CD274 (PD-L1), HLA-A, CXCL10, and CD163, displayed relatively high expression, suggesting a more inflamed tumor microenvironment. By contrast, group 1 was characterized by overall lower expression of immune activation and checkpoint molecules, with only isolated upregulation of selected transcripts, such as FOXP3 and CXCL10. The expression of regulatory mediators, such as TGFB1 and FOXP3, also varied between groups, indicating differences in immunoregulatory balance.

Due to the retrospective nature and incomplete quantitative metrics (coverage depth, CNA values, RNA QC), a full per-case integrated oncoprint table could not be generated. Therefore, a simplified summary is provided in Supplementary Table S1.

## 4. Discussion

In the presented series, two patients died: both had IDH-wild-type tumors. The diagnosis of diffuse astrocytoma or oligodendroglioma (grade 2–3), traditionally based solely on histomorphological criteria, now requires the demonstration of the presence of the IDH1 or IDH2 mutation and, in the case of oligodendrogliomas, of the 1p/19q codeletion [3,4]. In our series, with the exception of the two circumscribed tumors, namely case 8 (ganglioglioma) and case 9 (pilocytic astrocytoma), two of the tumors initially classified as diffuse LGG were found to be IDH-wild type. Out of these two cases, one was diagnosed before 2020 and was therefore initially correctly classified as fibrillary astrocytoma, grade 2, without any further molecular testing. In IDH-wild-type diffuse astrocytic tumors, several authors have documented that the presence of at least one of the four genetic abnormalities (TERT promoter mutations, EGFR amplifications, and the combination of gain of chromosome 7 or loss of chromosome 10 [+7/−10]) is sufficient for an upgrade to grade 4 [5,15]. In the fifth edition of the WHO classification, these tumors are therefore classified as IDH-wild type glioblastomas even in the absence of the typical morphological criteria of this neoplasm (microvascular proliferation, spontaneous intratumoral necrosis). This would effectively exclude one of the cases from our study (as it was EGFR-mutated and therefore no longer considered “low grade”). Case 10, however, does not yet present the characteristics for an upgrade to grade 4, and, despite meticulous molecular investigations it has undergone, it still appears to be an unclassifiable neoplasm.

IDH mutations were first reported in 2008 and are a distinctive feature of diffuse adult gliomas generally referred to as “lower grade” [16]. Most IDH-mutant gliomas carry the IDH1:c.395G > A p.R132H mutation. This mutation is present in approximately 85% of these tumors, while other mutations reported are rare [17,18]. The IDH2 gene encodes a protein homologous to IDH1, and its mutations, which are much less frequent, have the same oncogenic significance [19]. In our case series, all IDH-mutant tumors carried the R132H mutation, and no IDH2 mutations were found.

IDH-mutant tumors display a gain of function of the respective IDH1 and IDH2 enzymes, leading to overproduction of the oncometabolite 2-hydroxyglutarate, which causes a hypermethylated cell phenotype and consequently silencing of critical mechanisms of cellular differentiation [20,21].

The genomic instability of IDH-mutant tumors is the basis of their neoplastic progression, which leads tumors to accumulate new mutations over time, including amplifications of the MYC and CCND2 oncogenes [22]. IDH-mutant astrocytomas often carry other defining mutations, including TP53 and ATRX. The inactivating ATRX mutation causes abnormal telomere maintenance and is mutually exclusive with the activating TERT promoter mutation. The latter is rare in astrocytomas, but common in oligodendrogliomas and glioblastoma.

IDH-mutant and 1p/19q-co-deleted oligodendrogliomas are the second tumor classified as diffuse lower-grade adult glioma and are characterized by the coexistence of an IDH1 or IDH2 mutation and the deletion of the entire 1p and 19q arms, as well as a characteristic morphological appearance. The vast majority of these tumors also carry a TERT promoter mutation, which is mutually exclusive with the ATRX mutation. TP53 is mutated less frequently in oligodendrogliomas than in astrocytomas.

In our series, TP53 was found to be mutated in four cases (three IDH-mutant astrocytomas and one IDH-mutant and 1p/19q co-deleted oligodendroglioma). ATRX was mutated in only one case (IDH-mutant astrocytoma), while no mutation of the TERT promoter was found. Known genetic abnormalities that lead to tumor progression and worsening prognosis include homozygous deletion of CDKN2A and/or CDKN2B, amplification of CDK4, homozygous deletion or mutation of RB1, amplification of PDGFRA, alterations of MET, amplifications of MYCN, and mutations of PIK3R1 and PIK3CA [23].

No deletion of CDKN2A or 2B was found.

CDK4 amplification was found in four cases: two cases of IDH-mutant astrocytomas, a case of IDH-mutant and 1p/19q co-deleted oligodendroglioma, and a case of IDH-wild-type diffuse astrocytic tumor, which ultimately has been reclassified as a grade 4 glioblastoma due to the presence of EGFR amplification. MET amplification was found in one case of ganglioglioma, while two cases carried amplifications of MYCN, including a case of IDH-mutant and 1p/19q co-deleted oligodendroglioma and a case of IDH-wild-type diffuse astrocytic tumor. No cases were found with RB1 mutation or deletion, PDGFRA amplification, or with PIK3R1 or PIK3CA mutations.

The additional variants observed in genes such as CHEK2, FGFR3, TET2, and ASXL1 are not considered canonical drivers of gliomagenesis. Given the tumor-only design of the assay, these alterations should be interpreted cautiously, as some may represent rare germline variants or variants of uncertain significance. In particular, RET p.G691S and FGFR4 p.G388R, initially detected at high frequency (they were present in, respectively, seven and six cases), were excluded from the somatic analysis because they correspond to common germline polymorphisms documented in gnomAD v2.1.

RET p.G691S is widely reported in the literature as a non-synonymous (germline) polymorphism in normal populations, but even more frequent in some tumor series [24]. In several studies, it has been proposed as a risk/penetration modifier because it can increase oncogenic activity in the presence of other mutations. In some cases, it has also been found to be a somatic variant. It is not a classic activating “hot spot” of the kinase domain like M918T [25]. There is experimental evidence that the G691S variant can enhance pro-oncogenic functions in cell lines, and, in at least one study, it has been associated with the increased survival and proliferation response of glial cells. Furthermore, the GDNF–GFRA/RET pathway has been implicated in glioma models and may contribute to resistance/initiation of aggressive phenotypes. However, most cases where RET is clearly “actionable” involve fusions or activating mutations of the kinase domain [26].

FGFR4 p.G388R is also a common germline single-nucleotide polymorphism that replaces glycine with arginine in the transmembrane region; numerous studies and meta-analyses associate it with increased risk/poor prognosis in various cancers. Functional data indicate that the Arg388 variant modulates signaling (e.g., it promotes the activation of STAT3 and other pro-migratory/invasiveness pathways). Recent literature has shown that FGFR4 can promote glioblastoma and that the Arg388 allele increases STAT3/EMT signaling in other tumors. Therefore, Arg388 may promote cancerous behavior of glial cells by enhancing their migration, invasion, and resistance. However, classically targetable driver FGFR mutations (e.g., fusions, mutations in the kinase domain) are more frequently considered suitable for targeted therapy [27].

In the context of gliomas, the literature suggests that the GDNF–RET pathway and FGFR4 signaling may contribute to progression and treatment resistance in subsets of brain tumors. However, clinical evidence that renders RET or FGFR4 “actionable” refers to activating fusions or mutations in the kinase domain, not germline polymorphisms. Therefore, without demonstrating that these variants are somatic, clonal, and associated with clear pathway activation (e.g., elevated p-STAT3/p-ERK), their clinical interpretation should be treated with caution: they may represent biological modifiers rather than directly targetable drivers. Further functional studies and verification that the mutations are somatic are needed to establish whether they could serve as predictive biomarkers of response to RET/FGFR inhibitors in clinical trials [28].

Due to the high exclusion rate and the retrospective nature of the dataset, selection bias cannot be excluded. Patients with insufficient archival tumor material or missing research consent were not included, which may limit representativeness. Further prospective trials would be required to obtain normal brain tissue, to further remove the biological background other than the somatic mutation of the glioma samples.

## 5. Conclusions

Collectively, these findings reinforce the role of targeted next-generation sequencing in confirming the core molecular hallmarks of diffuse gliomas and in guiding their classification according to the WHO CNS 2021 criteria. At the same time, they highlight the methodological limitations of tumor-only approaches, underlining the need for matched normal samples and functional validation to definitively ascertain the pathogenic role of non-canonical variants. The transcriptional heterogeneity observed in our series mirrors the molecular complexity of diffuse gliomas described in larger genomic datasets. The clustering of IDH1/ATRX-mutant cases supports the existence of a distinct expression profile, consistent with the biological underpinnings of the IDH-mutant astrocytoma subgroup, as defined in the WHO CNS 2021 classification. Conversely, the broader dispersion of IDH-wild-type or non-canonical cases underscores the biological variability within this category, which may reflect admixture of different molecular entities, technical limitations related to FFPE-derived RNA, or the presence of passenger transcriptional alterations. Importantly, the analysis confirms that even within a small cohort, RNA-based profiling can highlight subgroup-specific signatures that complement DNA-based mutational data. Nonetheless, given the limited sample size and the reliance on FFPE material, the results must be interpreted cautiously. Further validation in larger series, ideally integrating bulk RNA sequencing with single-cell or spatial transcriptomics, will be required to clarify whether the expression differences observed are robust biological signals or reflect technical artifacts and sampling variability.

## Figures and Tables

**Table 1:** Cohort characteristics.

Cohort Size		11
Gender	Males	9 (81.81%)
	Females	2 (18.18%)
Age at diagnosis	Years	40 (19–75)
Type of diagnosis	Incidental	3 (27.27%)
	Symptomatic	8 (72.73%)
Tumor location	Frontal	8 (72.73%)
	Parietal	2 (18.18%)
	Brainstem	1 (9.09%)
Extent of resection	GTR	6 (54.55%)
	STR	4 (36.36%)
	PR (biopsy)	1 (9.09%)
Adjuvant treatments	Chemoradiotherapy with Temozolomide	3 (27.27%)
	PCV protocol	1 (9.09%)
	Radiotherapy alone	2 (18.18%)
	None (or not available)	5 (45.45%)
Follow-up	Months	14.67 (4.7–92.07)
PFS (6 patients progressed)	Months	17.5 (1.8–50.3)

**Table 2:** Cases carrying mutations interpreted as pathogenic, likely pathogenic, drug-putative, risk factor, or of uncertain significance/with conflicting interpretation.

Case Id	Diagnosis and Grade	Gene	Mutation
1	Astrocytoma IDH-mutant, G2	RET	G691S
		IDH1	R132H
		CHEK2	R474H
		ALK	A1601T
		BLM	S1209T
		IGF1R	R511Q
		SLX4	R669H
2	Astrocytoma IDH-mutant, G2	IRS2	G1057D
		IDH1	R132H
		FGFR4	G388R
		APC	No match
		TP53	C238R
3	Oligodendroglioma IDH-mutant and 1p/19q co-deleted, G3	RET	G691S
		RET	R982C
		IDH1	R132H
		FGFR4	G388R
		RANBP2	V2787G
		RANBP2	D2828E
		ERBB3	K747N
		POLE	No match
		PALB2	L332H
		TP53	No match
		JAK3	V718L
4	Astrocytoma IDH-mutant, G2	IRS2	G1057D
		IDH1	R132H
		FGFR4	G388R
		ATM	L1420F
		TP53	R273C
		AXIN2	S762N
5	Diffuse astrocytic tumor IDH-wild type, G2	GATA2	F270C
		RET	G691S
		IRS2	G1057D
6	Astrocytoma IDH-mutant, G2	RET	G691S
		IDH1	R132H
		TP53	R273C
		ATRX	K1176M
7	Oligodendroglioma IDH-mutant and 1p/19q co-deleted, G3	RET	G691S
		IDH1	R132H
		BLM	P355R
8	Ganglioglioma	RET	G691S
		IRS2	G1057D
		SPTA1	G1497E
		BLM	N1393D
		CHEK2	R474H
		CHEK2	L174F
9	Pilocytic astrocytoma	IRS2	G1057D
		FGFR4	G388R
		DDR2	M441I
		ATM	D1853V
		ZFHX3	G585S
		AXIN2	S762N
10	Diffuse astrocytic tumor IDH-wild type, G2	FGFR4	G388R
		SPTA1	T1522A
		EGFR	G598V
		MEN1	R176Q
		POLE	G6R
		ERCC4	P379S
11	Astrocytoma IDH-mutant, G2	FGFR4	G388R
		CDC73	Q338P
		IDH1	R132H
		RET	G691S
		RET	R982C

**Table 3:** Genetic amplifications of each patient.

Case Id	Diagnosis and Grade	Amplifications
1	Astrocytoma IDH-mutant, G2	None
2	Astrocytoma IDH-mutant, G2	None
3	Oligodendroglioma IDH-mutant and 1p/19q co-deleted, G3	MYCN
		MYC
		CCND1
		FGF19
		ATM
		CDK4
		FGF14
4	Astrocytoma IDH-mutant, G2	CDK4
5	Diffuse astrocytic tumor IDH-wild type, G2	None
6	Astrocytoma IDH-mutant, G2	None
7	Oligodendroglioma IDH-mutant and 1p/19q co-deleted, G3	MDM4
		MYC
8	Ganglioglioma	CDK6
		MET
		BRAF
9	Pilocytic astrocytoma	FGF1
		CDK6
		BRAF
10	Diffuse astrocytic tumor IDH-wild type, G2	MDM4
		MYCN
		EGFR
		CDK6
		BRAF
		CDK4
11	Astrocytoma IDH-mutant, G2	CDK4

## Data Availability

The data will only be made available from the corresponding author upon reasonable request.

## Supplementary Materials

The following supporting information can be downloaded at https://www.mdpi.com/article/10.3390/jcm15010053/s1.

## Abbreviations

The following abbreviations are used in this manuscript:

BRAF	B-Raf proto-oncogene, serine/threonine kinase
CNS	Central nervous system
EGFR	Epithelial Growth Factor Receptor
FLAIR	Fluid-attenuated inversion recovery
LGG	Low-grade glioma
MRI	Magnetic resonance imaging
NGS	Next-Generation Sequencing
OS	Overall survival
PFS	Progression-free survival
TERT	Telomerase Reverse Transcriptase

## References

[1] Ostrom Q.T., Cioffi G., Gittleman H., Patil N., Waite K., Kruchko C., Barnholtz-Sloan J.S. (2019). CBTRUS Statistical Report: Primary Brain and Other Central Nervous System Tumors Diagnosed in the United States in 2012–2016. Neuro-Oncology.

[2] Louis D.N., von Deimling A. (2017). Grading of diffuse astrocytic gliomas: Broders, Kernohan, Zülch, the WHO… and Shakespeare. Acta Neuropathol..

[3] Louis D.N., Wesseling P., Brandner S., Brat D.J., Ellison D.W., Giangaspero F., Hattab E.M., Hawkins C., Judge M.J., Kleinschmidt-DeMasters B. (2020). Data Sets for the Reporting of Tumors of the Central Nervous System: Recommendations From The International Collaboration on Cancer Reporting. Arch. Pathol. Lab. Med..

[4] Gonzalez Castro L.N., Wesseling P. (2020). The cIMPACT-NOW updates and their significance to current neuro-oncology practice. Neuro-Oncol. Pract..

[5] Louis D.N., Perry A., Wesseling P., Brat D.J., Cree I.A., Figarella-Branger D., Hawkins C., Ng H.K., Pfister S.M., Reifenberger G. (2021). The 2021 WHO Classification of Tumors of the Central Nervous System: A summary. Neuro-Oncology.

[6] Brat D.J., Aldape K., Colman H., Holland E.C., Louis D.N., Jenkins R.B., Kleinschmidt-DeMasters B.K., Perry A., Reifenberger G., Stupp R. (2018). cIMPACT-NOW update 3: Recommended diagnostic criteria for “Diffuse astrocytic glioma, IDH-wildtype, with molecular features of glioblastoma, WHO grade IV”. Acta Neuropathol..

[7] Horbinski C., Miller C.R., Perry A. (2011). Gone FISHing: Clinical lessons learned in brain tumor molecular diagnostics over the last decade. Brain Pathol..

[8] Al-Kateb H., Knight S.M., Sivasankaran G., Voss J.S., Pitel B.A., Blommel J.H., Jerde C.R., Rumilla K.M., Lee J.L., Mattson N.R. (2025). Clinical Validation of the TruSight Oncology 500 Assay for the Detection and Reporting of Pan-Cancer Biomarkers. J. Mol. Diagn..

[9] Froyen G., Geerdens E., Berden S., Cruys B., Maes B. (2022). Diagnostic Validation of a Comprehensive Targeted Panel for Broad Mutational and Biomarker Analysis in Solid Tumors. Cancers.

[10] Dal Bo M., Polano M., Ius T., Di Cintio F., Mondello A., Manini I., Pegolo E., Cesselli D., Di Loreto C., Skrap M. (2023). Machine learning to improve interpretability of clinical, radiological and panel-based genomic data of glioma grade 4 patients undergoing surgical resection. J. Transl. Med..

[11] Chen S., Liu M., Huang T., Liao W., Xu M., Gu J. (2018). GeneFuse: Detection and visualization of target gene fusions from DNA sequencing data. Int. J. Biol. Sci..

[12] Stupp R., Mason W.P., van den Bent M.J., Weller M., Fisher B., Taphoorn M.J.B., Belanger K., Brandes A.A., Marosi C., Bogdahn U. (2005). Radiotherapy plus concomitant and adjuvant temozolomide for glioblastoma. N. Engl. J. Med..

[13] Weller M., van den Bent M., Preusser M., Le Rhun E., Tonn J.C., Minniti G., Bendszus M., Balana C., Chinot O., Dirven L. (2021). EANO guidelines on the diagnosis and treatment of diffuse gliomas of adulthood. Nat. Rev. Clin. Oncol..

[14] Wen P.Y., Bent M.v.D., Youssef G., Cloughesy T.F., Ellingson B.M., Weller M., Galanis E., Barboriak D.P., de Groot J., Gilbert M.R. (2023). RANO 2.0: Update to the Response Assessment in Neuro-Oncology Criteria for High- and Low-Grade Gliomas in Adults. J. Clin. Oncol..

[15] Tesileanu C.M.S., Dirven L., Wijnenga M.M.J., Koekkoek J.A.F., Vincent A.J.P.E., Dubbink H.J., Atmodimedjo P.N., Kros J.M., Van Duinen S.G., Smits M. (2020). Survival of diffuse astrocytic glioma, IDH1/2 wildtype, with molecular features of glioblastoma, WHO grade IV: A confirmation of the cIMPACT-NOW criteria. Neuro-Oncology.

[16] Parsons D.W., Jones S., Zhang X., Lin J.C.-H., Leary R.J., Angenendt P., Mankoo P., Carter H., Siu I.-M., Gallia G.L. (2008). An integrated genomic analysis of human glioblastoma multiforme. Science.

[17] Balss J., Meyer J., Mueller W., Korshunov A., Hartmann C., von Deimling A. (2008). Analysis of the IDH1 codon 132 mutation in brain tumors. Acta Neuropathol..

[18] Watanabe T., Nobusawa S., Kleihues P., Ohgaki H. (2009). IDH1 mutations are early events in the development of astrocytomas and oligodendrogliomas. Am. J. Pathol..

[19] Yan H., Parsons D.W., Jin G., McLendon R., Rasheed B.A., Yuan W., Kos I., Batinic-Haberle I., Jones S., Riggins G.J. (2009). IDH1 and IDH2 mutations in gliomas. N. Engl. J. Med..

[20] Dang L., White D.W., Gross S., Bennett B.D., Bittinger M.A., Driggers E.M., Fantin V.R., Jang H.G., Jin S., Keenan M.C. (2009). Cancer-associated IDH1 mutations produce 2-hydroxyglutarate. Nature.

[21] Turcan S., Rohle D., Goenka A., Walsh L.A., Fang F., Yilmaz E., Campos C., Fabius A.W.M., Lu C., Ward P.S. (2012). IDH1 mutation is sufficient to establish the glioma hypermethylator phenotype. Nature.

[22] Brat D.J., Verhaak R.G., Aldape K.D., Yung W.K., Salama S.R., Cooper L.A., Rheinbay E., Miller C.R., Vitucci M., Cancer Genome Atlas Research Network (2015). Comprehensive, Integrative Genomic Analysis of Diffuse Lower-Grade Gliomas. N. Engl. J. Med..

[23] Brat D.J., Aldape K., Colman H., Figrarella-Branger D., Fuller G.N., Giannini C., Holland E.C., Jenkins R.B., Kleinschmidt-DeMasters B., Komori T. (2020). cIMPACT-NOW update 5: Recommended grading criteria and terminologies for IDH-mutant astrocytomas. Acta Neuropathol..

[24] Taşkan T., Noori F., Kurukahvecioğlu O., Karaman N., Gönenç A. (2025). Neurturin gene IVSI-663 polymorphism but not RET variants is associated with increased risk for breast cancer. Lab. Med..

[25] Elisei R., Cosci B., Romei C., Bottici V., Sculli M., Lari R., Barale R., Pacini F., Pinchera A. (2004). RET exon 11 (G691S) polymorphism is significantly more frequent in sporadic medullary thyroid carcinoma than in the general population. J. Clin. Endocrinol. Metab..

[26] Yu Z., Li H., Wang M., Luo W., Xue Y. (2022). GDNF regulates lipid metabolism and glioma growth through RET/ERK/HIF-1/SREBP-1. Int. J. Oncol..

[27] Gabler L., Jaunecker C.N., Katz S., van Schoonhoven S., Englinger B., Pirker C., Mohr T., Vician P., Stojanovic M., Woitzuck V. (2022). Fibroblast growth factor receptor 4 promotes glioblastoma progression: A central role of integrin-mediated cell invasiveness. Acta Neuropathol. Commun..

[28] Colombo C., Minna E., Rizzetti M.G., Romeo P., Lecis D., Persani L., Mondellini P., Pierotti M.A., Greco A., Fugazzola L. (2015). The modifier role of RET-G691S polymorphism in hereditary medullary thyroid carcinoma: Functional characterization and expression/penetrance studies. Orphanet J. Rare Dis..

