# Supplementary material for: Molecular Characterization of Adult-Type Lower-Grade Glioma (WHO Grade 1–3) with Targeted Next-Generation Sequencing: A Retrospective, Single-Institution Experience
Source: J Clin Med. 2025 Dec 21;15(1):53. doi: 10.3390/jcm15010053 (PMC12786670; doi:10.3390/jcm15010053)
Supplement: Supplementary file 1 [file jcm-15-00053-s001.zip › jcm-3942686-supplementary.pdf]

Description of additional information Additional Methods

Additional Tables

Table S1

Table S2

Table S3

Table S4

Table S5

Table S1 Clinical characteristics, molecular findings, and outcomes of the 11 FFPE low-grade glioma cases included in the study. The table summarizes demographic data (sex, age), 2021 WHO diagnosis, MGMT promoter methylation status when available, key pathogenic or likely pathogenic variants identified through TSO500 DNA sequencing, and clinical follow-up information. Progression-free survival (PFS) and overall survival (OS) are reported in months. "N/A" indicates data not available; "unmet." indicates absence of methylation of MGMT promoter; "met." indicates methylation of MGMT promoter.

Case id	Sex	Age	WHO 2021 diagnosis	MGMT status	Key pathogenic/likely pathogenic variants	Follow up (months)	PFS	OS
1	Male	31	astrocytoma IDH-mutant, G2	N/A	none	34,97	NA	NA
2	Male	47	astrocytoma IDH-mutant, G2	N/A	APC, TP53 (C238R)	79,80	50,37	NA
3	Male	37	oligodendroglioma IDH-mutant and 1p/19q codeleted, G3	N/A	TP53	75,73	NA	NA
4	Male	40	astrocytoma IDH-mutant, G2	unmet.	TP53	39,07	37,90	NA
5	Male	75	diffuse astrocytic tumor IDH-wild type, G2	N/A	none	14,67	7,17	14,73
6	Male	33	astrocytoma IDH-mutant, G2	N/A	TP53	11,60	NA	NA
7	Female	48	oligodendroglioma IDH-mutant and 1p/19q codeleted, G3	N/A	none	4,70	2,67	NA
8	Male	25	ganglioglioma	N/A	none	4,70	NA	NA
9	Male	19	pilocytic astrocytoma	N/A	none	92,07	1,97	NA
10	Female	75	diffuse astrocytic tumor IDH-wild type, G2	N/A	EFGR (G598V)	6,33	6,17	8,63
11	Male	46	astrocytoma IDH-mutant, G2	met.	none	6,00	NA	NA

Table S2 RNA-seq quality control metrics for all samples. Reported metrics include GC content (%), total number of raw paired-end reads (millions), percentage of bases removed during trimming, and post-trimming read counts and GC content. These metrics were generated using FastQC (v0.11.x) and the nf-core/rnaseq workflow v3.17.0.

Sample	Mean Raw GC (%)	Mean Raw Reads (M)	Mean Trimmed Bases (%)	Mean Post-trim GC (%)	Mean Post-trim Reads (M)
1	40.0	37.1	1.95	40.0	35.9
2	40.0	43.3	1.30	40.0	42.7
3	41.0	41.2	1.10	41.0	40.7
4	41.5	28.4	5.85	41.0	24.6
5	39.0	45.6	3.50	39.0	42.6
6	44.0	36.3	1.30	44.0	35.9
7	42.0	26.2	3.10	42.0	24.7
8	42.0	43.9	1.05	42.0	43.5
9	40.0	41.4	0.95	40.0	41.1
10	41.5	39.4	0.90	41.0	39.1
11	40.0	71.7	0.70	40.0	71.5

Table S3 Summary of GeneFuse fusion detection results across all 11 FFPE-derived RNA-seq samples. No biologically meaningful gene fusion was identified in the cohort. Ten of eleven cases showed zero fusion events. A single sample (9) displayed a low-confidence ALK–ROS1 fusion-like signal supported by only 7 reads (6 unique). Detailed inspection demonstrated that this represented a false-positive artifact rather than a true genomic rearrangement. The event showed insufficient read support, unstable alignment with soft clipping and mismatches at the breakpoint, non-canonical transcript orientation, lack of reproducibility across repeated runs, and no orthogonal evidence in BAM alignments or expression profiles. These features are consistent with RNA fragmentation–related artifacts typical of aged FFPE tissue. Thus, no actionable or biologically interpretable fusion was detected in any of the 11 samples.

Sample ID	Fusion Calls	ALK–ROS1	Read Support	Interpretation
1	0	No	–	No fusion detected
2	0 (only intragenic artifacts)	No	2–4	FFPE artifact
3	0	No	–	No fusion detected
4	0	No	–	No fusion detected
5	0	No	–	No fusion detected
6	0	No	–	No fusion detected
7	0	No	–	No fusion detected
8	0	No	–	No fusion detected
9	1	Yes (false)	7 total / 6 unique	False positive artifact
10	0	No	–	No fusion detected
11	0	No	–	No fusion detected

Table S4 Detailed characteristics of the reads underlying the false ALK–ROS1 fusion-like signal in sample 5009. GeneFuse reported a small cluster of reads with breakpoints at positions 66–77 and diff values between 0 and 3. All reads share an almost identical internal genomic sequence, without a true junction between two different genes, and show low-level mismatches and soft clipping at the breakpoint. These features, together with the very limited read support (7 total, 6 unique) and the absence of orthogonal confirmation, indicate that this represents a FFPE-related alignment artifact rather than a genuine ALK–ROS1 fusion.

Read ID (short)	Break position	diff (left | right)	Read direction	Comment
NB552559:264:HGKCGBGXW:1:22211:25626:12398	77	(1 | 1)	reversed complement	Repeated internal genomic sequence; no true gene–gene junction
NB552559:264:HGKCGBGXW:4:22611:3889:2439	72	(1 | 3)	original	Same breakpoint motif; low-level micro-homology artifact
NB552559:264:HGKCGBGXW:4:13408:22893:6252	72	(1 | 3)	original	Sequence pattern identical to other reads
NB552559:264:HGKCGBGXW:2:11109:2513:14445	72	(1 | 3)	original	Fragmented FFPE RNA; unstable mapping at junction
NB552559:264:HGKCGBGXW:4:23601:17119:10391	66	(0 | 3)	original	Minimal support; no evidence of a bona fide ALK–ROS1 fusion

Table S5 Coverage distribution across detected somatic variants (VAF >5%). Summary statistics of read depth at 8,036 variant positions with variant allele frequency >5%. The median coverage was 215× (IQR: 148–431×), with all variants meeting the minimum threshold of 100× depth, ensuring high confidence in variant calling.

Coverage Metric	Value
Number of Variants	8,036
Mean Depth (×)	393.6
Median Depth (×)	215.0
Minimum Depth (×)	100
Maximum Depth (×)	20,913
Standard Deviation	976.7
25th Percentile (×)	148.0
75th Percentile (×)	431.0

Abbreviations: IQR, interquartile range; VAF, variant allele frequency
